# Survival and adaptation of *Streptococcus phocae* in host environments

**DOI:** 10.1371/journal.pone.0296368

**Published:** 2024-01-30

**Authors:** Daniela Numberger, Ursula Siebert, Peter Valentin Weigand

**Affiliations:** 1 Institute for Microbiology, University of Veterinary Medicine Hannover, Foundation, Hannover, Germany; 2 Institute for Terrestrial and Aquatic Wildlife Research, University of Veterinary Medicine Hannover, Foundation, Buesum, Germany; Wrocław University of Environmental and Life Sciences: Uniwersytet Przyrodniczy we Wroclawiu, POLAND

## Abstract

Marine mammals are sentinel species representing the “health” of our oceans on which we are dependent. There are many threats to marine mammals including infectious diseases that increase with climate change and pollution of the marine environment. *Streptococcus phocae* has frequently been isolated from diseased or dead marine mammals. However, its pathogenicity and contribution to disease in marine mammals is still unknown. As bacteria including (potential) pathogens has to deal with different host environments during colonization or infection, we investigated the survival of *S*. *phocae* in fresh porcine and phocid blood, in seawater and in the presence of macrophages and (epithelial) cells from harbor seals and pigs. Furthermore, we tested adherence on and invasion of different (marine) mammalian cells by *S*. *phocae*. Our results showed that *S*. *phocae* can survive in seawater for at least 11 and 28 days at 16°C and 4°C, respectively. It is able to grow in blood of harbor and grey seals, but not in porcine blood. Furthermore, *S*. *phocae* is adherent and invasive to cells from seals and pigs, while the portion of invasive cells was higher in seal derived cells. Macrophages of harbor seals were more efficient in killing *S*. *phocae* than porcine macrophages. Our results indicate that *S*. *phocae* has strategies enabling it to adapt to the marine environment and seal hosts.

## Introduction

The marine ecosystem is challenged by many factors such as anthropogenic pollution, fisheries, shipping, offshore-constructions, harmful algal blooms, acidification and climate change [1–7]. These factors can be stressors for marine mammals, considered as sentinels for the marine ecosystem [8, 9], and might lead to a higher susceptibility for infectious diseases through suppressive effects on their immune system [6, 10].

Beta-hemolytic streptococci are frequently found in marine mammals and are known to have pathogenic potential (for review see Numberger et al., 2021 [11]). One of the predominant species is *Streptococcus phocae* [11] that was first isolated from dead harbor seals in Norway 1988 suffering mostly from pneumonia [12]. In the following years, *S*. *phocae* was still mainly isolated from marine mammals including cape fur seal [13], grey seal [14], ringed seal, harbor porpoise [15], Caspian seal [16], California sea lion [17], short-beaked common dolphin [18], spotted seal [19], bottlenose dolphin [20], white whale [21], stellar sea lion [22], Guadalupe fur seal, elephant seal [23] and southern sea otters [24]. To our knowledge there is no isolation of *S*. *phocae* from healthy marine mammals reported. A recent publication describes the first isolation of *S*. *phocae* from a terrestrial mammal, the mink (*Neovison vison*) [25]. It was isolated from three unrelated cases of farmed minks suffered from pneumonia and dermal infection with lesions. Genome sequences revealed a close relation to harbor seal isolates suggesting a possible cross-species transmission, where fish as diet might serve as a vector [25]. The subspecies *S*. *phocae* subsp. *salmonis* is a severe pathogen of Atlantic salmon causing disease and lethality [26], but differs from harbor seal isolates described as *S*. *phocae* subsp. *phocae*. While the contribution of *S*. *phocae* in infectious disease of pinnipeds and cetacean is largely unknown, *S*. *phocae* infections in sea otters of Alaska were significantly associated with a high morbidity and mortality [24]. In the Kachemak Bay and Resurrection Bay, Alaska, where many northern sea otters died from strep syndrome, *S*. *phocae* could be detected via PCR in seawater and mussel samples [27]. The authors also observed a high presence of harbor seals in the Kachemak Bay and suggested a possible association between sea otter and harbor seal infections [27]. These data are based on PCR and do not allow any conclusions on the survival rate of *S*. *phocae* in seawater and its tolerance to high salinity and low temperatures.

Despite many reports on the wide distribution/occurrence of *S*. *phocae* in marine mammals, particularly pinnipeds, little is known about its persistence in the host and the marine environment or the possible transmission between hosts. In general, pathogenic and commensal bacteria of marine mammals are challenged by different host environments and thus need different adaptation strategies for survival and colonisation in various host niches. Such host environments include (mucosal and epithelial) cells, blood, macrophages and also seawater, which might play an important role in the transmission of bacteria between marine hosts. The first barrier are epithelial cells, to which infecting bacteria need to adhere in order to colonize the host. For dissemination, bacterial pathogens may invade the cells or tissue in order to translocate into the blood stream. The ability of a pathogen to survive in host blood is a prerequisite for a successful systemic infection and is unknown for *S*. *phocae*. In the tissue, resident immune cells are crucial for bacterial clearance. For *S*. *pyogenes* it has been described that tissue resident macrophages [28] and dendritic cells [29] play a crucial role to limit streptococcal dissemination and initiate protective immune defences. Marine mammals have direct contact to the marine environment including the seawater. Thus, having marine mammals as host seawater is another environment that *S*. *phocae* needs to challenge with, e.g. for transmission through seawater.

In this study we investigated the behaviour of three different *S*. *phocae* isolates from harbor seal, grey seal and harbor porpoise in the three aforementioned “host environments” of harbor seals, including seawater, seal cells, blood and monocyte-derived macrophages (MDM) as immune-active cells. We incubated three different isolates of *S*. *phocae* in seawater from the German North Sea at 4°C and 16°C and inoculated fresh blood of harbor seals. Additionally, we co-incubated *S*. *phocae* with monocyte-derived macrophages from harbor seals. The results were compared to experiments in the porcine system as a terrestrial mammal to see adaptations to the marine host.

## Material and methods

### *Streptococcus phocae* and *Streptococcus suis* isolates

*Streptococcus phocae* isolates were collected during examinations of dead harbor seals (*Phoca vitulina*), grey seals (*Halichoerus grypus*) and harbor porpoises (*Phocoena phocoena*) found during 1996–1999 (**Table 1**). All isolates were identified as *S*. *phocae* by sequencing and MALDI TOF MS Microflex LT/SH (Biotyper) with MBT Compass Library revision L (Nov. 2020; Bruker Corporation, MA, USA). *Streptococcus suis*, *serotype 2 was kindly provided by H*. *Smith (Lelystad*, *Netherlands* [30]*) S*. *phocae* and *S*. *suis* were cultured in BD Bacto™ Todd Hewitt Broth (THB, Becton Dickinson, Heidelberg, Germany) or on Columbia agar plates supplemented with 7% (*v/v*) sheep blood (Oxoid^TM^, Thermo Fisher Scientific, Waltham, MA, USA). All experiments were done with *S*. *phocae* stocks take from the log phase.

**Table 1:** Information of *Streptococcus phocae* isolates used in this study.

Isolate	Species	Organ	Year	Place (Germany)	Lance-field	Hemolysis
Sp1	*P*. *vitulina*	lung	1996	Pellworm	F	β
Sp16	*H*. *grypus*	lung	1998	St. Peter-Ording	F	β
Sp55	*P*. *phocoena*	bowel lymphnode	1999	Sylt	F	β

### Sampling and preparation of seawater and blood from harbor seals

North Sea Water was collected in sterile 2 L glass bottles at the coast of Büsum, Germany and transported to the lab at 4°C. Seawater was filtered through 0.22 μm filters for sterilization without changing the chemical or physical properties and stored at 4°C until use.

Fresh blood samples were taken from healthy harbor and grey seals at the seal rehabilitation station in Friedrichskoog, Germany during medical examination and transported to the lab in Lithium-Heparin S-Monovettes^®^ (Sarstedt, Nümbrecht, Germany) within 3 hours at 4°C. The medical examination was part of the preparation for releasing the animals to the wild after successful rehabilitation. No animals were harmed or sacrificed. Porcine blood was provided by the Clinic for Swine and Small Ruminants, Forensic Medicine and Ambulatory Service, University of Veterinary Medicine Hannover, Foundation as part of a practical teaching course.

### Incubation of *S*. *phocae* isolates in seawater and blood samples

For testing the persistence of *S*. *phocae* in seawater, 100 ml of sterile-filtered seawater were inoculated with 10^6^ colony forming units (CFU). Positive controls were run in THB and sterile-filtered seawater was tested negatively for the presence of *S*. *phocae* and any other microorganisms. To test different temperatures, the inoculated samples and controls were incubated at 4°C and 16°C. Experiments were repeated three times (biological replicates). Every hour or on a daily basis subsamples were taken for plating in triplicates (technical replicates).

For each *S*. *phocae* isolate 1 ml of fresh blood from harbor seals (n = 3), grey seals (n = 3) or pigs (n = 3) were inoculated with 10^6^ CFU and incubated at 37°C while rotating. Every hour subsamples were taken for plating in triplicates (technical replicates). Controls in 1 ml THB were incubated and plated in parallel. Blood without bacterial inoculum was plated as negative control.

As all three *S*. *phocae* isolates were not able to grow in seawater and decreased over time when incubating in seawater, we were interested if the salinity could be a limiting factor. So, we checked the salt tolerance of one of the *S*. *phocae* isolates (Sp55) in comparison to the non-marine *Streptococcus suis*. To do so, we used THB medium, in which both streptococcal species can grow and added different concentration of sodium chloride. The average salinity of the North Sea is 35 PSU (practical salinity unit and the average salinity of the Baltic Sea is 18 PSU. As we can find harbor seals in both marine environments, we tested these two salinities and added 18 g/l = 18 PSU or 35 g/l = 35 PSU sodium chloride to THB medium. After sterilization the two bacterial species were incubated in triplicates at 37°C in 50 ml tubes and CFU was determined by plating subsamples on blood agar plates after three, six and 24 hours. *S*. *suis* was also incubated at 4°C in seawater from the North Sea and checked daily by plating a subsample in comparison to only THB. Statistical analyzes to test for significant differences between the strains were performed by using unpaired t-test (p < 0.05) in Prism 9 by GraphPad Software Version 9.0.0 (121).

### Isolation of monocytes

Peripheral blood mononuclear cells (PBMC) from harbor seals and pigs were isolated from fresh heparin blood according to Reichel et al., 2015 [31]. After dilution of heparinized blood with PBS (phosphate buffered saline; Sigma-Aldrich, Taufkirchen, Germany) containing 0.02% EDTA (1:3 ratio; Carl Roth, Karlsruhe, Germany), 20 mL of a Biocoll^®^ separating solution (1.077 g/ml; Bio&SELL GmbH, Nürnberg, Germany) was used to separate 12 mL of diluted blood by centrifugation (800 ×g for 45 min, 4°C). The lymphocyte/monocyte layer was collected and washed three times in RPMI 1640 medium (Gibco, Thermo Fisher Scientific, Waltham, MA, USA) (400 ×g, 10 min, 4°C). Phocid cells from 12 mL blood were seeded into a 75 cm^2^ tissue plastic flask (Greiner Bio-One, Frickenhausen, Germany), previously coated with 2% sterile gelatin (Carl Roth, Karlsruhe, Germany) solution followed by autologous harbor seal plasma and allowed to adhere in RPMI 1640 medium supplemented with 10% FCS (fetal calf serum), 1% penicillin/streptomycin (Sigma-Aldrich, Taufkirchen, Germany). For porcine cells uncoated flasks were used. Cells were incubated at 37°C and 5% CO_2_. Non-adherent cells were removed after 24 h and adherent monocytes were supplied with fresh medium. After one week of differentiation, monocyte-derived macrophages (MDM) were used for subsequent experiments. An unpaired t-test (p < 0.05) was used for statistical analyzes (GraphPad Prism Version 9.0.0 (121)).

### Killing assay with monocyte-derived macrophages (MDMs)

Adherent MDM, isolated from porcine or seal blood, were washed twice with PBS and detached using accutase (PAN-Biotech GmbH, Aidenbach, Germany) following the manufacturer’s protocol. The cells were resuspended in RPMI 1640 medium and counted using a Neubauer chamber. MDM were infected with a multiplicity of infection (MOI) of 10:1 by co-incubating 2.5 × 10^6^ bacterial CFU/ml of each *S*. *phocae* isolate with 2.5 × 10^5^ MDM cells/ml in RPMI 1640 medium (*w/o* antibiotics). The experiments were run in triplicates. Subsamples were plated on blood agar plates in triplicates every hour for CFU counting. A control of bacteria was run in RPMI 1640 medium without MDM. Data were tested for statistical significance by using unpaired t-test (p < 0.05) in Prism 9 by GraphPad Software Version 9.0.0 (121).

### Immortalized cell lines and primary cells

Cell lines of the kidney (SEK2b) and dermis (SED) of a harbor seal were kindly provided by Dr. Matthias König from the Friedrich Löffler Institute, Germany. The cells were obtained from a dead harbor seal dissected in the seal breeding station Friedrichskoog, Germany and immortalized via transfection of the SV40 T-antigen and the pSV3neo plasmid [32, 33]. New-born pig tracheal epithelial cells (NPTr) were kindly provided by F. Meurens (Nantes, France) [34]. Primary cells were obtained from kidneys of freshly dead harbor seals (pvPRC) or harbor porpoises (ppPRC) necropsied at the Institute for Terrestrial and Aquatic Wildlife research, University of Veterinary Medicine Hannover, Foundation, Büsum, Germany. This was part of a monitoring program that is funded by Schleswig-Holstein, Germany and approved by the responsible ministry. No animals were harmed or sacrificed for this study. Fresh pieces of the kidney were incubated for 48–72 hours at 4°C in Dulbecco’s Modified Eagle Medium (DMEM; high glucose; Gibco, Thermo Fisher Scientific, Waltham, MA, USA) supplemented with 0.1 mg/ml Penicillin-Streptomycin, 2.5 μg/ml amphotericin B, 1 mg/ml protease and 10 μg/ml DNase (Sigma-Aldrich Chemie GmbH, Taufkirchen, Germany). The kidney was cut in a sterile petri dish into small pieces (~ 0.5 cm^3^) and a 50 mL tube was prepared with 50 mL DMEM supplemented with heat-inactivated FCS to which a cell strainer (100 μm) was applied to collect the renal cells. The small pieces of the kidney were disrupted by mincing and washing them through the cell strainer by using the plunger of a syringe. The cells were then centrifuged at 250 ×g for 5 min at 4°C. In addition, the medium in which the kidney had been incubated for 48–72 hours was also centrifuged to collect potential detached cells. The supernatants were discharged, and the cell pellets were washed twice with cold phosphate buffered saline (PBS, Sigma-Aldrich, Taufkirchen, Germany). Following, the cell pellets were resuspended in Renal Epithelial Cell Medium 2 (RECM, PromoCell GmbH, Heidelberg, Germany) supplemented with 10% (*v/v*) FCS + 0.1 mg/ml Penicillin-Streptomycin, 2.5 μg/ml amphotericin B and 50 μg/ml gentamicin (Carl Roth, Karlsruhe, Germany) and incubated in a cell culture dish (Ø 150 mm; Sarstedt, Nümbrecht, Germany) for 2 hours at 37°C and 5% CO_2_ to allow fibroblasts to adhere. The supernatants were finally transferred to collagen I (Sigma-Aldrich, Taufkirchen, Germany)-coated T-75 cell culture flasks (Sarstedt, Nümbrecht, Germany) in RECM medium including supplements mentioned above. Medium was changed after 24 hours and then every 2 days. If required, primary cells were detached and split using a cell scraper.

### Infection of mammalian cells with *S*. *phocae*

Mammalian cells were seeded on 24-well plates and infected with the three different *S*. *phocae* isolates (**Table 1**) at MOI = 5 in duplicates (technical replicates). Each infection was repeated three times for biological replicates. Infected cells were incubated at 37°C and 5% CO_2_. For double immune fluorescence (DIF) infection was additionally performed on glass cover slips (thickness 1 round, Ø: 12 mm; Carl Roth, Karlsruhe, Germany). Each sample that was taken for CFU counting was diluted serially and several dilutions were plated in triplicates on blood agar plates. For determining the bacterial growth, the supernatant was plated at 0, 3, 6 and 20 hours post infection (p.i.). To get the CFU of adherent plus invasive bacteria, the cells were washed three times with PBS, detached with 1× Trypsin-EDTA (Gibco, Thermo Fisher Scientific, Waltham, MA, USA) and lysed with 1% saponin (Carl Roth, Karlsruhe, Germany). The lysed cell suspension was again plated for CFU counting. Invasive bacteria only were quantified by lysis after extracellular antibiotic treatment with 100 μg/mL penicillin and 100 μg/mL gentamicin and plated for CFU counting. Data were statistically analyzed using GraphPad Prism version 8.0.1 for Windows (GraphPad Software, San Diego, CA, USA). Figures show means and standard errors (SEM). Statistical significance was determined by unpaired, nonparametric Mann-Whitney test unpaired t-test using Prism 9 by GraphPad Software Version 9.0.0 (121) (p < 0.05 was considered statistically significant).

### Double-Immune-Fluorescence (DIF) microscopy

Coverslips with adhered infected cells were washed with PBS and blocked with 5% FCS for 20 min at room temperature. Then, the coverslips were again washed three times with PBS and incubated with the primary polyclonal rat antibody against *S*. *phocae* (1:100, Davids Biotechnology, Regensburg, Germany) for 90 min in a dark humid chamber. After washing the coverslips three times with PBS the Alexa Fluor® 488 goat-anti-rat IgG secondary antibody (1:1000, Invitrogen, Thermo Fisher Scientific, Waltham, MA, USA) was added and incubated for 30 min in a dark humid chamber. Another three wash steps in PBS were followed by permeabilization of mammalian cells with 0.2% Triton® X 100 (Carl Roth, Karlsruhe, Germany) for 5 min. Then, intracellular bacteria were stained by repeating the steps above, but using Alexa Fluor® 568 goat-anti-rat IgG secondary antibody (1:1000, Invitrogen, Thermo Fisher Scientific,Waltham, MA, USA) to distinguish between adherent and invasive bacteria. After washing three times in PBS, nuclei were stained by 0.5 μg/ml DAPI (Cell Signaling Technology, Leiden, Netherlands) for 10 min and cover slips were mounted with ProLong^®^ Gold antifade reagent (Cell Signaling Technology, Leiden, Netherlands) at 4°C overnight. Microscopy of infected cells was performed with a Nikon Eclipse Ti-S microscope equipped with a Nikon DS-QiMC-U2 camera controlled by NIS-Elements BR version 4.51.01 (Nikon, Tokyo, Japan). For display purposes, images were identically adjusted for contrast and brightness using Fiji-ImageJ version 1.53c.

## Results

### Persistence of *S*. *phocae* in seawater at 4 and 16°C

**Fig 1:**
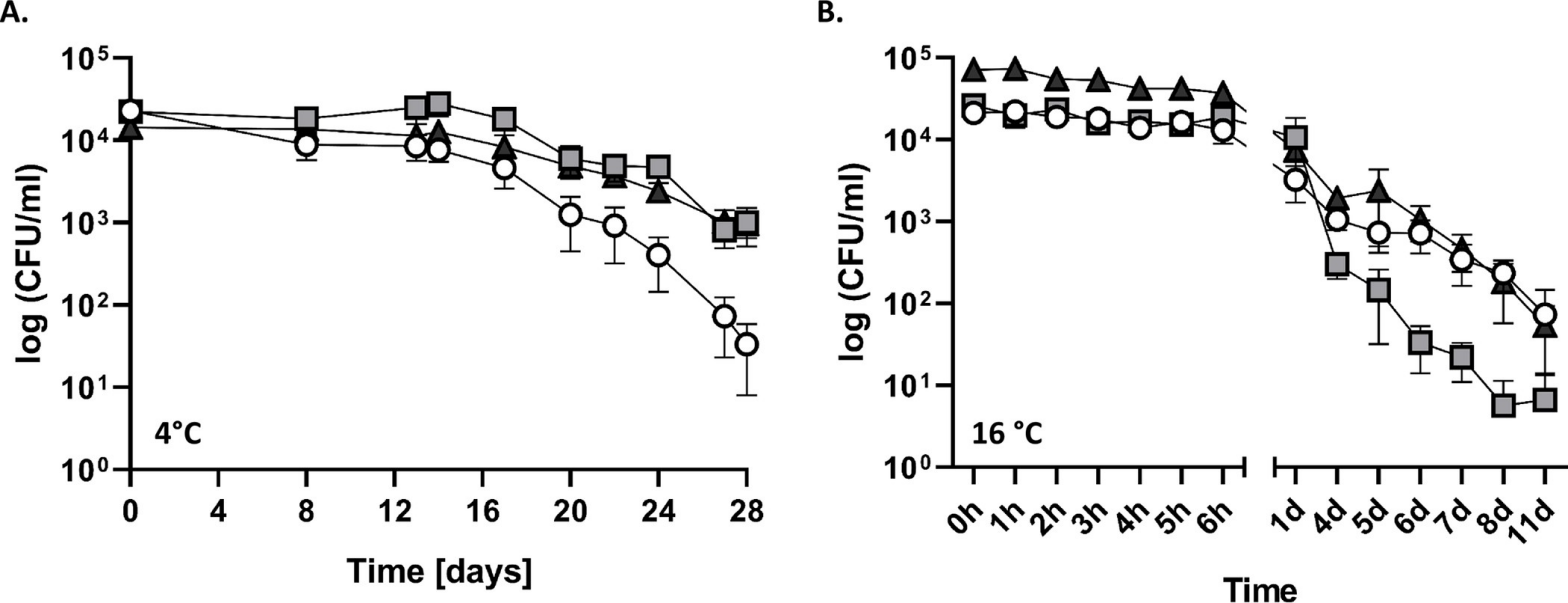
Persistence of *S*. *phocae* in seawater at 4°C (A.) and 16°C (B.). 100 ml of sterile-filtered seawater from the North Sea was inoculated with 10^6^ CFU of three *S*. *phocae* isolates Sp1 (white circle), Sp16 (light grey square) and Sp55 (dark grey triangle). Graphs show the mean with standard error of mean (SEM).

As a colonizer/pathogen of marine mammals it is inevitable that *S*. *phocae* is exposed to seawater. Thus, we tested the survival rate of *S*. *phocae* in seawater at 4°C and 16°C using sterile-filtered Seawater from the North Sea, Germany. At 4°C, *S*. *phocae* was still culturable after 28 days (**Fig 1A**). The first 17 days all *S*. *phocae* isolates were stable at ~ 10^4^ CFU/ml and decreased from day 17 to day 28 to an average of 9.9 × 10^2^ CFU/ml (Sp16, Sp55) and 3.3 × 10^1^ CFU/ml (Sp1). *S*. *phocae* survived in seawater at 16°C for at least 11 days while a decrease in viability could be observed starting from 24 hours on (**Fig 1B**). The first 6 hours all three isolates were quite stable at an average of 3.0 × 10^4^ CFU/ml, respectively. From day 1 to day 11 all three *S*. *phocae* isolates decreased from an average of 7.1 × 10^3^ CFU/ml to an average of 4.4 × 10^1^ CFU/ml, respectively. Statistical analyzes showed that all three isolates behave similarly in seawater, as there were no significant differences.

The salt tolerance of *S*. *phocae* was tested in Todd Hewitt Broth supplemented with 18 g/l NaCl (18 PSU) and 35 g/l (35 PSU) and compared to a non-marine streptococcal species, the porcine pathogen *S*. *suis*. As there were no differences between the *S*. *phocae* isolates, we only used Sp55 for the comparison to *S*. *suis*. While *S*. *phocae* was growing in 18 and 35 PSU, *S*. *suis* was not able to grow. Also, *S*. *suis* did not survive as long as *S*. *phocae* in seawater at 4°C but died within 14 days (**S1 Fig**).

### Survival and growth of *S*. *phocae* in porcine and seal blood

**Fig 2:**
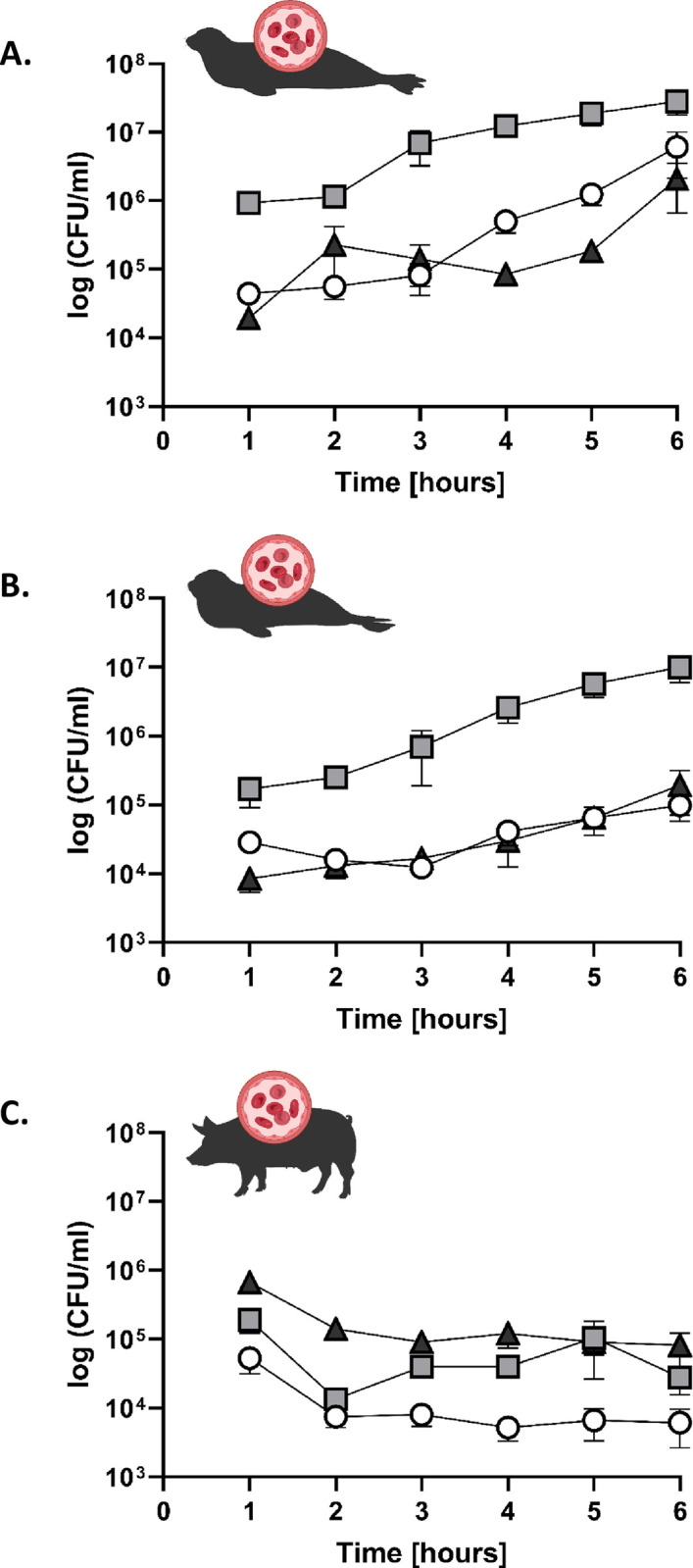
Incubation of *S*. *phocae* in blood in harbor seals (A.), grey seals (B.) and pigs (C.). 1 ml of heparin blood was inoculated with 10^6^ CFU of three *S*. *phocae* isolates Sp1 (white circle), Sp16 (light grey square) and Sp55 (dark grey triangle). Graphs show the mean with standard error of mean (SEM).

Blood is a host environment that many pathogens are exposed to during their dissemination. Therefore, we analyzed the survival of *S*. *phocae* in blood from harbor seals, grey seals and compared it with the survival in porcine blood as a terrestrial mammal. All three *S*. *phocae* isolates showed growth in both harbor seal and grey seal blood increasing two magnitudes, but not in porcine blood (**Fig 2**). It was able to increase from average 3.3 × 10^5^ CFU/ml to 1.2 × 10^7^ CFU/ml within 6 hours in harbor seal blood and from average 6.9 × 10^4^ CFU/ml to 3.4 × 10^6^ CFU/ml in grey seal blood. In porcine blood *S*. *phocae* showed a drop within the first two hours and was then stable from two to six hours at an average of 5.2 × 10^4^ CFU/ml. No significant differences between the three isolates were observed. Besides, a drop of bacterial concentration within the first hour could be observed as a result of acclimatization of bacteria. The inoculation dose was hence a “target dose” od 10^6^ CFU. As it was not relevant for showing the growth of *S*. *phocae*, we decided to remove that data points from **Fig 2**.

### Co-incubation of *S*. *phocae* with monocyte-derived macrophages (MDM)

When entering the host, *S*. *phocae* might be attacked by the host immune system.

**Fig 3:**
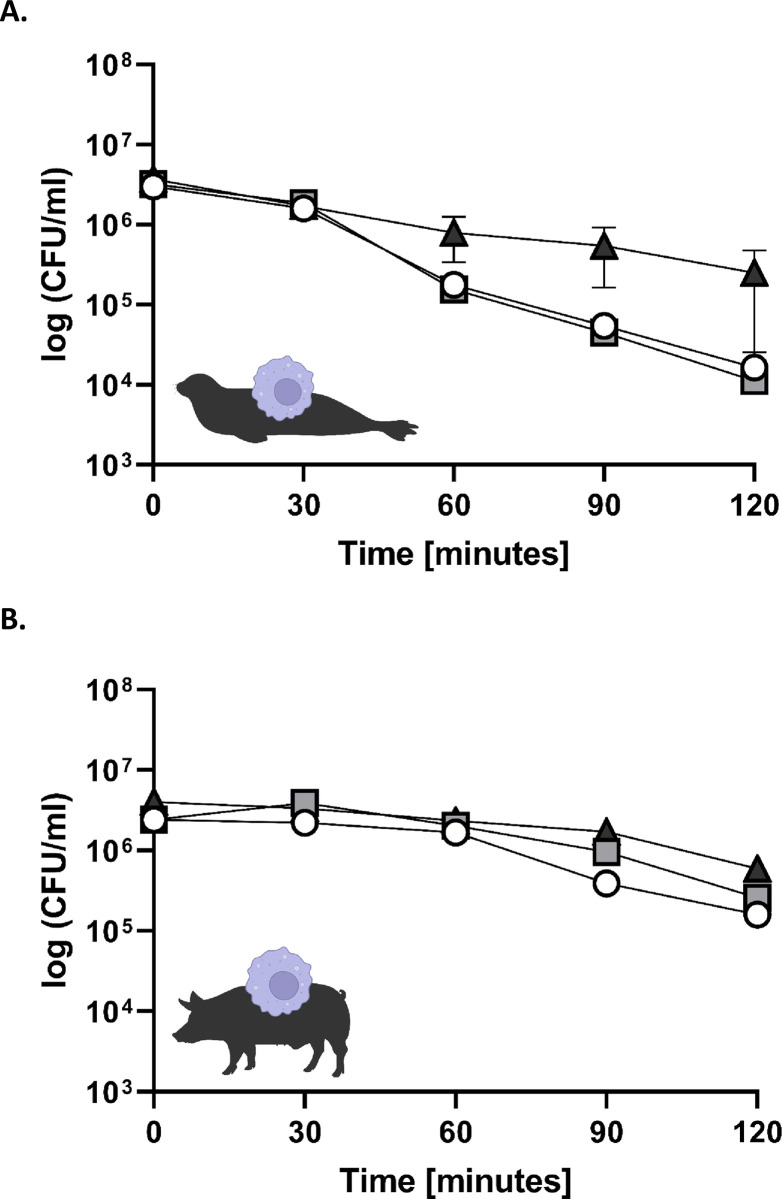
Killing assay with *S*. *phocae* and monocyte-derived macrophages (MDM) from harbor seals and pigs. MDM were co-incubated with three *S*. *phocae* isolates Sp1 (white circle), Sp16 (light grey square) and Sp55 (dark grey triangle) at MOI = 10. Graphs show the mean with standard error of mean (SEM).

To investigate, if *S*. *phocae* is killed by phocid and porcine macrophages, we isolated monocytes from blood of seals and swine. After differentiation to (monocyte-derived) macrophages (MDM), they were incubated with the three *S*. *phocae* isolates Sp1, Sp16 and Sp55 in RPMI 1640 medium (10% FCS, *w/o* antibiotics). The CFU of *S*. *phocae* decreased within 120 minutes independent of the host origin (seal or swine) (**Fig 3**). However, a stronger decrease could be observed with seal MDM in comparison to porcine MDM. While seal MDM cause a decrease of two magnitudes from average 3.3 × 10^6^ CFU/ml to 1.7 × 10^4^ CFU/ml, *S*. *phocae* decreased only one magnitude with porcine MDM (average 2.9 × 10^6^ CFU/ml to 3.4 × 10^5^ CFU/ml). Again, no significant differences between the three isolates were observed as revealed by unpaired t-test.

### Adherence to and invasion of mammalian cells

**Fig 4:**
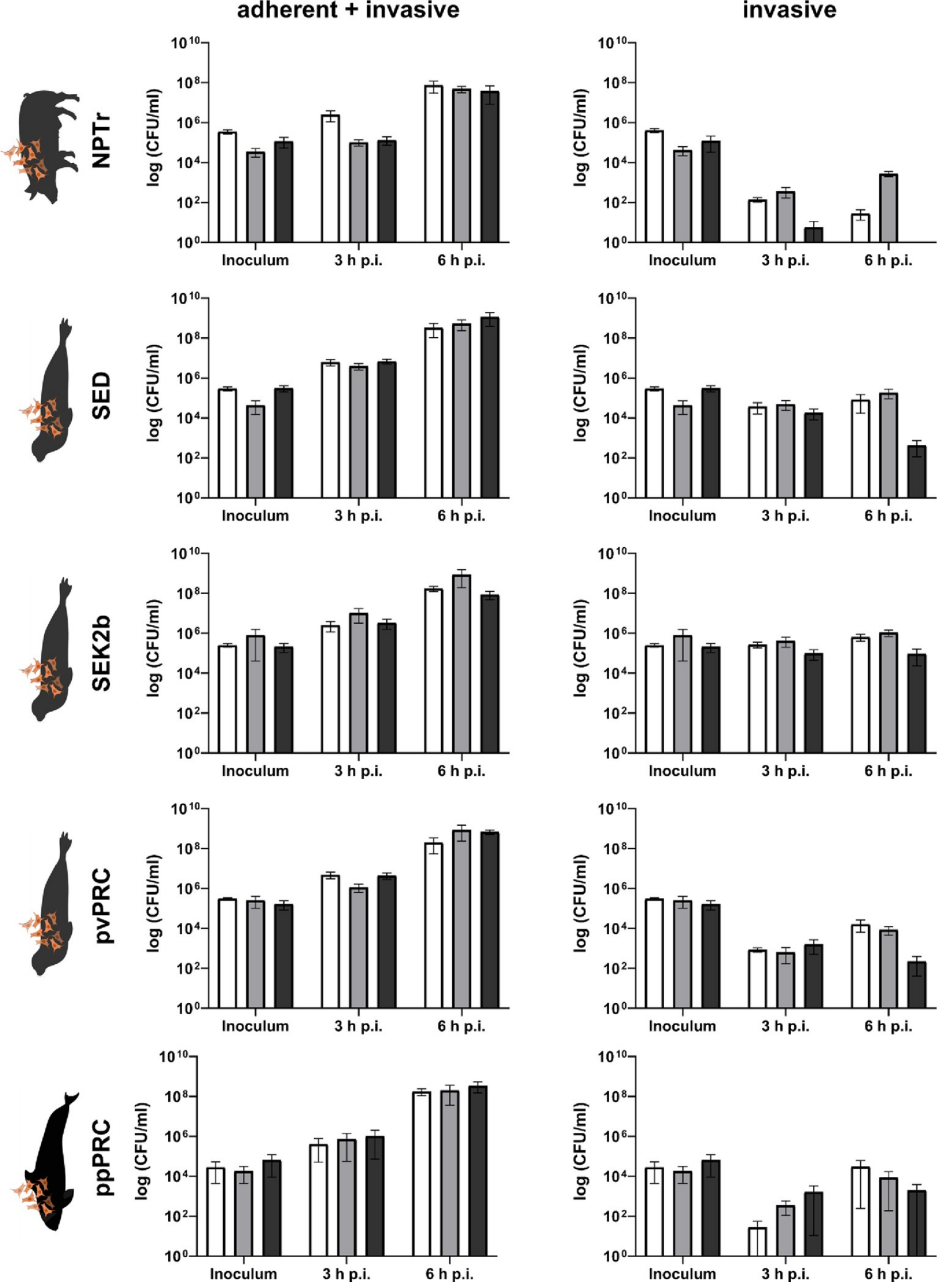
Adherence and invasion of *S*. *phocae* to different (marine) mammalian cells. Cells were infected with three *S*. *phocae* isolates Sp1 (white), Sp16 (light grey) and Sp55 (dark grey) at MOI = 5. Bars show the mean with standard error of mean (SEM). NPTr (new-born pig trachea cells); SED (seal dermis cells); SEK2b (seal kidney cells); pvPRC (*Phoca vitulina* primary renal cells); ppPRC (*Phocoena phocoena* primary renal cells). The data at 20 h p.i. are not shown, because the cells started to die. No significant differences could be detected using GraphPad Prism version 8.0.1 for Windows (GraphPad Software, San Diego, CA, USA) and unpaired, nonparametric Mann-Whitney test.

Pathogens are able to adhere to and sometimes to invade epithelial (mucosal) cells. To test the adherence and invasion of *S*. *phocae* to cells from the dermis and kidney of harbor seals and compared it to the infection of a porcine cell line, the NPTr (newborn pig trachea) cells. The infection experiments demonstrated that the three *S*. *phocae* isolates Sp1, Sp16 and Sp55 were both adherent and invasive in all tested cell types (**Fig 4**). In all experiments, the CFU of bacteria associated with cells (adherent and invasive) increase from three to six hours, while in most cases the number of invasive bacteria alone differ only slightly from three to six hours. Statistical analyzes did not show any significant differences between the three *S*. *phocae* isolates. However, the highest number of invasive bacteria was observed in the seal dermis (SED) and seal kidney (SEK2b) cell lines and the lowest number in the NPTr cell line. Statistical analyzes did not reveal significant differences. However, the p-value for invasive bacteria in NPTr versus SEK2b cells was 0.0569, so quite close to significance. The highest number of invasive bacteria in NPTr cells was Sp16 with 3.6 × 10^3^ CFU/ml at 6 hours, while the SEK2b cells had up to 1.5 × 10^6^ CFU/ml and the SED cells up to 4.9 × 10^5^ CFU/ml invasive bacteria at six hours p.i.

Double-immunofluorescence analysis is based on antibody staining of extracellular bacteria followed by staining of intracellular bacteria after permeabilization. While intracellular bacteria were shown with red fluorescence only, some of the adherent bacteria showed a yellowish staining due to the double-staining and overlapping of red and green fluorescence. It supported the results on adherence and invasion by showing a clear distinction of adherent and invasive bacteria (**S2 Fig**).

## Discussion

In this study we investigated the survival in and adaptation of *S*. *phocae* to seal host environments to which the bacteria are exposed during colonization and invasion. For this, we tested the survival of *S*. *phocae* in blood and seawater, investigated bacterial adherence to and invasion of host epithelial cells and analyzed phagocytic killing by monocyte-derived macrophages. For comparison to a terrestrial mammal we used porcine blood and host cells, respectively.

In comparison to the swine pathogen *S*. *suis*, *S*. *phocae* was able to grow in Todd Hewitt Broth supplemented with 35 g/L NaCl (35 PSU) indicating a high salt tolerance and an adaptation to the marine environment. Furthermore, *S*. *phocae* was able to survive at least 11 and 28 days in seawater at 16°C and 4°C, respectively. This shows that the survival rate in the marine environment increases with lower water temperatures confirming an adaptation to the marine environment another adaptation, as most pinnipeds and many other marine mammals live in temperate to polar regions [35]. Bacteria are known to reduce their metabolic activity at lower temperatures to protect themselves against cold shock [36, 37]. We assume that growth of *S*. *phocae* was limited by nutrients in our experiment, as a tendency for longer survival was observed in the THB control. Thus, *S*. *phocae* might be able to persist for even longer times in the marine environment, where the availability of nutrients might be better through natural nutrient cycles or plankton and algal blooms [38, 39]. To conclude, the transmission of *S*. *phocae* through seawater is a possible scenario including filtrating organisms (e.g. mussels) that might accumulate *S*. *phocae* and are the prey of several marine mammalian species such as sea otters [40, 41]. Further studies are required to identify factors that influence the survival rate of *S*. *phocae* such as particle association and high nutrient concentrations. It is known that bacteria including fecal bacteria have higher survival rates in sediment than in water [42] as well as when associated with particles [43, 44] demonstrating the protective features of those environments. In addition, particles and sediment often have higher concentrations of nutrients and organic matter and thus, may increase survival rates [45–47]. This could also be true for *S*. *phocae*. A recent study could detect *S*. *phocae* in 37% of nasopulmonary mite samples (family *Halarachnidae*) from southern sea otters and California sea lions indicating those mites as potential vector for bacterial pathogens [48]. Moreover, the association with zooplankton has protective and promotive features for bacteria including pathogens, which again might enhance the bacterial survival rate [49].

The results of the incubation of *S*. *phocae* in fresh seal blood showed that it is able to grow within 6 hours, indicating pathogenic potential after entering the seal host bloodstream. In contrast, *S*. *phocae* could not grow in porcine blood, but persisted for at least 6 hours. This indicates an adaptation to the marine mammalian host or seals in particular, e.g. by enhanced strategies for immune evasion in the marine host. The streptococcal M-protein, for instance, is known to bind host fibrinogen as immune evasion strategy by inhibiting the complement system [50, 51]. This might be one of many processes that are host specific as it has been shown for canine fibrinogen-binding proteins of *Staphylococcus pseudintermedius* [52].

Co-incubation with phocid and porcine monocyte-derived macrophages showed a clear killing effect of *S*. *phocae*. One explanation could be that *S*. *phocae* is able to evade in the blood by binding host proteins such as fibrinogen, which is not present in co-incubations with only MDM. Another possible evasion factor of many streptococcal species is the antiphagocytic capsule of streptococci, which can also depend on complement factors in the host blood [53, 54]. The multiplication of encapsulated *S*. *suis* in murine blood is restricted by the host complement system, as shown by a significantly higher multiplication rate in wild-type blood than in blood lacking the complement factor C3 [54]. In the presence of S. suis antiserum, no multiplication could be observed [54]. In another study authors could show that 48% of bovine mammary macrophages ingested encapsulated *Streptococcus uberis* with a killing rate of 35% and after opsonization of *S*. *uberis* with homologous antiserum it was 84% and 52%, respectively [53]. Further studies are necessary including co-cultivation with other blood components such as neutrophils and the complement system. The stronger decrease of *S*. *phocae* with seal MDM in comparison to porcine MDM could be an adaptation of the seal host to *S*. *phocae* infections and thus, be more efficient in killing *S*. *phocae* in contrast to porcine MDM. The MDM of the two different hosts might have different receptors for the recognition of *S*. *phocae*, as it has been shown for complement receptors of murine and human MDM and the pathogen *Salmonella typhi* [55].

Infection experiments with *S*. *phocae* investigated the adherence to and invasion in different host epithelial cells. We did not observe any significant differences by statistical tests. However, there was a higher number of invasive bacteria in seal derived cells, particularly SEK2b cells than in the porcine NPTr cells. An unpaired t-test revealed a p-value of 0.0569, which is close to significance. Nonetheless, further studies are necessary to confirm these differences. Although adherence is mainly mediated by specific adhesins and other mechanisms that might be host-dependent [56, 57], passive and host-independent hydrophobic interactions might play a role in unspecific adherence [58]. In contrast, invasion needs active interaction with the host-cell and might therefore be host-dependent and–specific, e.g. receptor binding [59]. Host-specificity of *S*. *phocae* was also demonstrated in infection experiments of Atlantic salmon (*Salmo salar*) by González-Contreras et al. [60], where only the *S*. *phocae* isolate from salmon, not from seal caused mortality or pathological signs in fish. Subsequently, two subspecies were described based on more differences between salmon and seal isolates, *Streptococcus phocae* subsp. *salmonis* and *S*. *phocae* subsp. *phocae*, respectively [61]. A role as a commensal is also possible as it has been demonstrated for *S*. *suis* showing pathogenic and commensal behavior [62]. However, data on the prevalence of *S*. *phocae* in healthy individuals are still missing and therefore should be investigated in further studies.

Our data suggest the idea of a co-evolution of *S*. *phocae* and the seal host. *S*. *phocae* seems to be adapted to the seal by its ability to proliferate in seal blood and enhanced invasion mechanisms of cells from seals than pigs. The seal host showed a more efficient killing of *S*. *phocae* by macrophages than the porcine host. Following studies should focus on the mechanisms behind our experiments to support and explain our findings.

## Supporting information

S1 Fig Incubation of *Streptococcus suis* and *S*. *phocae* Sp55 in Todd Hewitt Broth (THB) supplemented with 35 g/l (= 35 PSU) or 18 g/l (= 18 PSU) sodium chloride at 37°C to test for salt tolerance between the two streptococcal species [A+B]. A control of THB without salt (= 0 PSU) was included [C]. The growth of *S*. *suis* in sterile-filtered seawater from the North Sea and THB as a control was additionally investigated [D]. Experiments were run in triplicates. PSU = practical salinity unit (1 PSU = 1g /l).
(PDF)

S2 Fig Double-immune fluorescence of different mammalian cells infected with *S*. *phocae*. Only three samples are shown representing the three situations for a qualitative evaluation of the adherence and invasion behaviour of *S*. *phocae*: adherent (green/yellowish) and invasive (red) bacteria (first row, NPTr cells, Sp16, 3 h p.i.); only invasive (red) bacteria (second row, ppPRCs, Sp16, 20 h p.i.) and only adherent (green/yellowish) bacteria (third row, SED cells, Sp16, 6 h p.i.).The first column shows the nucleus of eukaryotic cells by the blue fluorescence of DAPI. The second column shows the adherent bacteria by the green or fluorescence of the second antibody Alexa Fluor® 488 goat-anti-rat IgG. The third column shows the invasive bacteria by the red fluorescence of the second antibody Alexa Fluor® 568 goat-anti-rat IgG. The fourth column shows the merged channels including differential interference contrast (DIC) capture to see the cell body. Scale bar 10 μm. All images original magnification 600×.
(PDF)

S1 Graphical abstract (TIF)
