# Supplementary material for: Survival and adaptation of Streptococcus phocae in host environments
Source: PLoS One. 2024 Jan 30;19(1):e0296368. doi: 10.1371/journal.pone.0296368 (PMC10826952; doi:10.1371/journal.pone.0296368)
Supplement: S1 Fig — Incubation of Streptococcus suis and S. phocae Sp55 in Todd Hewitt Broth (THB) supplemented with 35 g/l (= 35 PSU) or 18 g/l (= 18 PSU) sodium chloride at 37°C to test for salt tolerance between the two streptococcal species [A+B]. A control of THB without salt (= 0 PSU) was included [C]. The growth of S. suis in sterile-filtered seawater from the North Sea and THB as a control was additionally investigated [D]. Experiments were run in triplicates. PSU = practical salinity unit (1 PSU = 1g /l). (PDF) [file pone.0296368.s001.pdf]

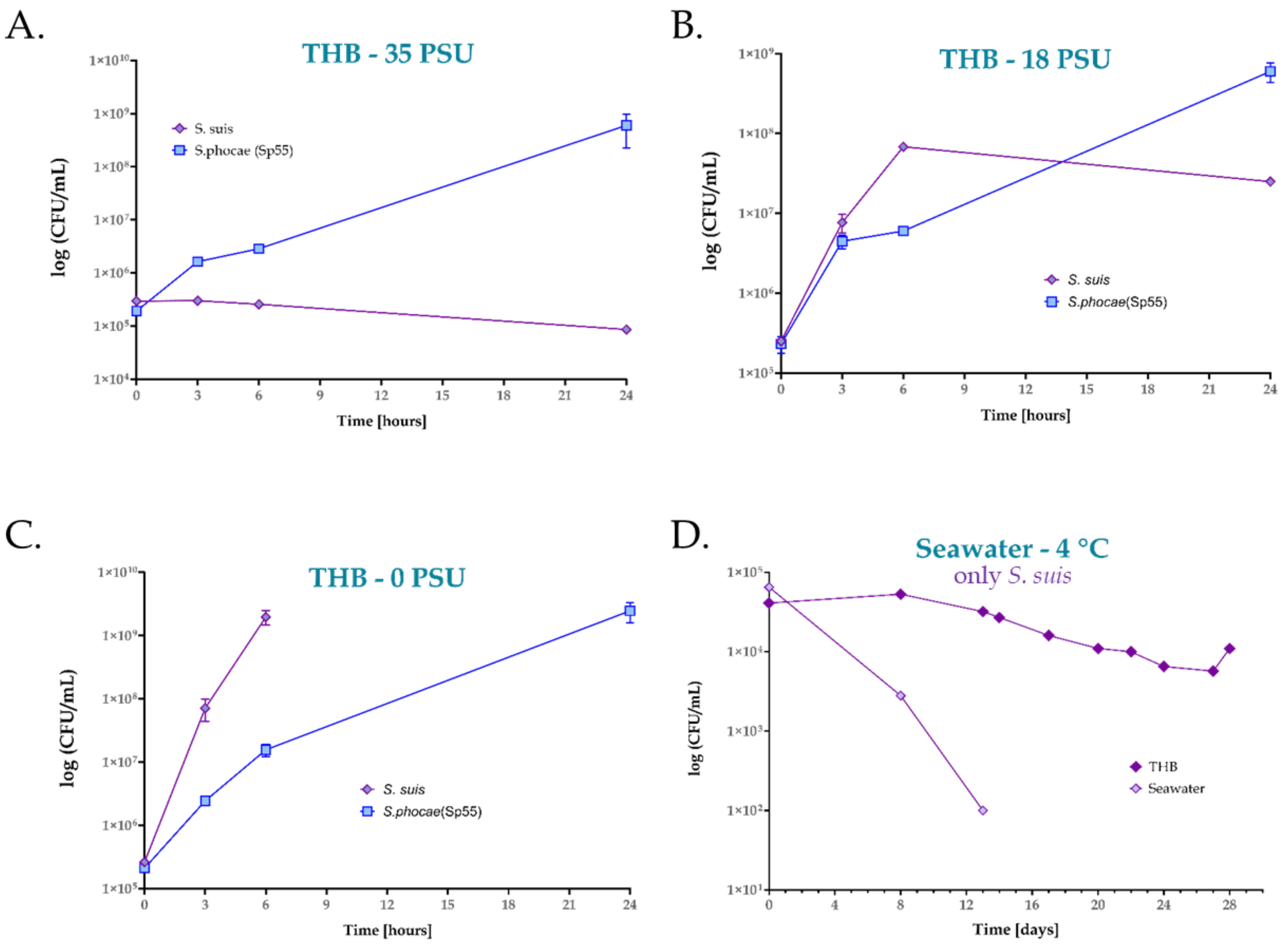

S1 Figure: Incubation of *Streptococcus suis* and *S. phocae* Sp55 in Todd Hewitt Broth (THB) supplemented with 35 g/l (= 35 PSU) or 18 g/l (= 18 PSU) sodium chloride at 37°C to test for salt tolerance between the two streptococcal species [**A+B**]. A control of THB without salt (= 0 PSU) was included [**C**]. The growth of *S. suis* in sterile-filtered seawater from the North Sea and THB as a control was additionally investigated [**D**]. Experiments were run in triplicates. PSU = practical salinity unit (1 PSU = 1g /l).
