# Supplementary material for: Survival and adaptation of Streptococcus phocae in host environments
Source: PLoS One. 2024 Jan 30;19(1):e0296368. doi: 10.1371/journal.pone.0296368 (PMC10826952; doi:10.1371/journal.pone.0296368)
Supplement: S2 Fig — Only three samples are shown representing the three situations for a qualitative evaluation of the adherence and invasion behaviour of S. phocae: adherent (green/yellowish) and invasive (red) bacteria (first row, NPTr cells, Sp16, 3 h p.i.); only invasive (red) bacteria (second row, ppPRCs, Sp16, 20 h p.i.) and only adherent (green/yellowish) bacteria (third row, SED cells, Sp16, 6 h p.i.).The first column shows the nucleus of eukaryotic cells by the blue fluorescence of DAPI. The second column shows the adherent bacteria by the green or fluorescence of the second antibody Alexa Fluor® 488 goat-anti-rat IgG. The third column shows the invasive bacteria by the red fluorescence of the second antibody Alexa Fluor® 568 goat-anti-rat IgG. The fourth column shows the merged channels including differential interference contrast (DIC) capture to see the cell body. Scale bar 10 μm. All images original magnification 600×. (PDF) [file pone.0296368.s002.pdf]

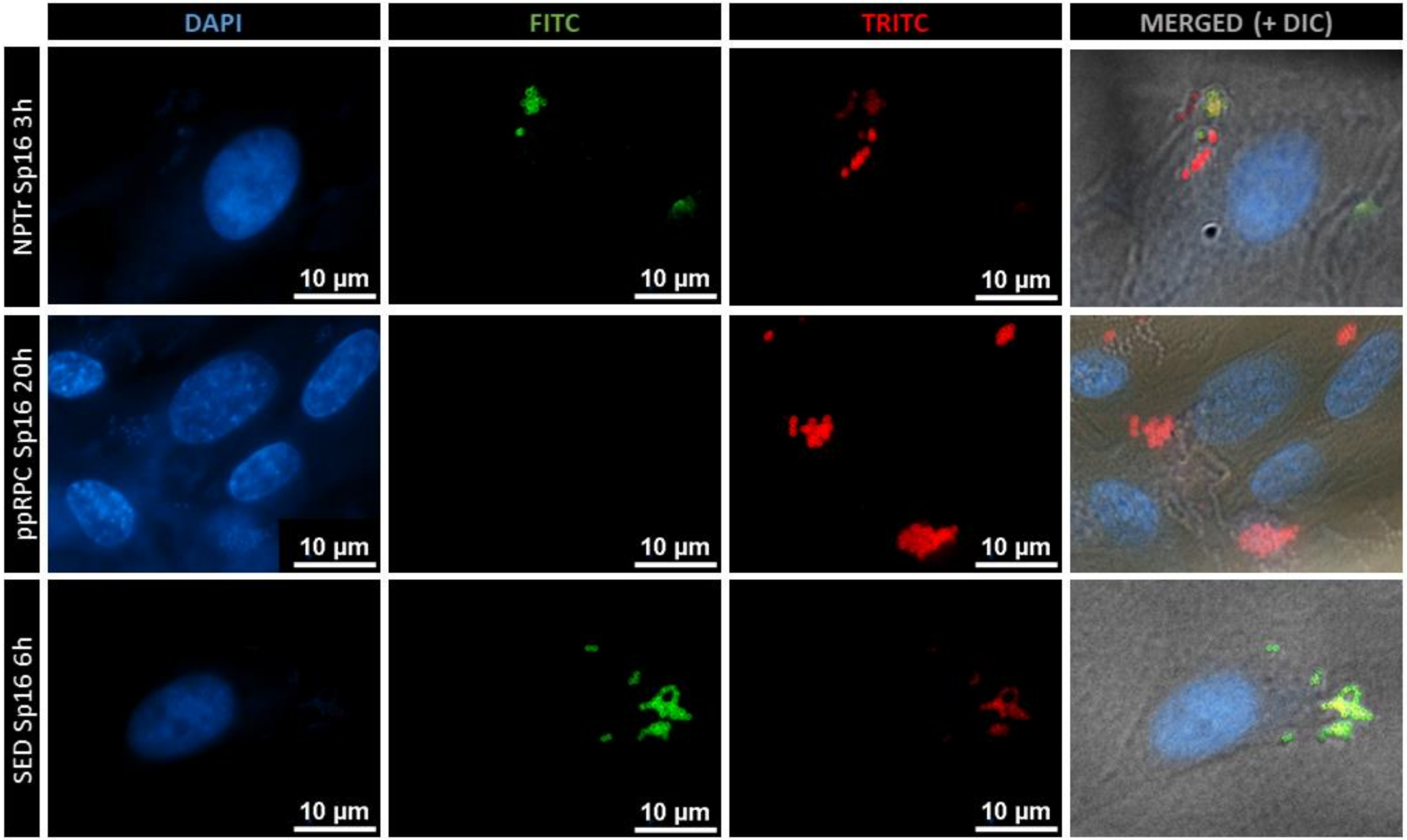

S2 Figure: Double-immune fluorescence of different mammalian cells infected with *S. phocae*. Only three samples are shown representing the three situations for a qualitative evaluation of the adherence and invasion behaviour of *S. phocae*: adherent (green/yellowish) and invasive (red) bacteria (first row, NPTr cells, Sp16, 3 h p.i.); only invasive (red) bacteria (second row, ppPRCs, Sp16, 20 h p.i.) and only adherent (green/yellowish) bacteria (third row, SED cells, Sp16, 6 h p.i.).The first column shows the nucleus of eukaryotic cells by the blue fluorescence of DAPI. The second column shows the adherent bacteria by the green or fluorescence of the second antibody Alexa Fluor® 488 goat-anti-rat IgG. The third column shows the invasive bacteria by the red fluorescence of the second antibody Alexa Fluor® 568 goat-anti-rat IgG. The fourth column shows the merged channels including differential interference contrast (DIC) capture to see the cell body. Scale bar 10 µm. All images original magnification 600×.
