# Supplementary figures and images for: Survival and adaptation of Streptococcus phocae in host environments
Source: PLoS One. 2024 Jan 30;19(1):e0296368. doi: 10.1371/journal.pone.0296368 (PMC10826952; doi:10.1371/journal.pone.0296368)

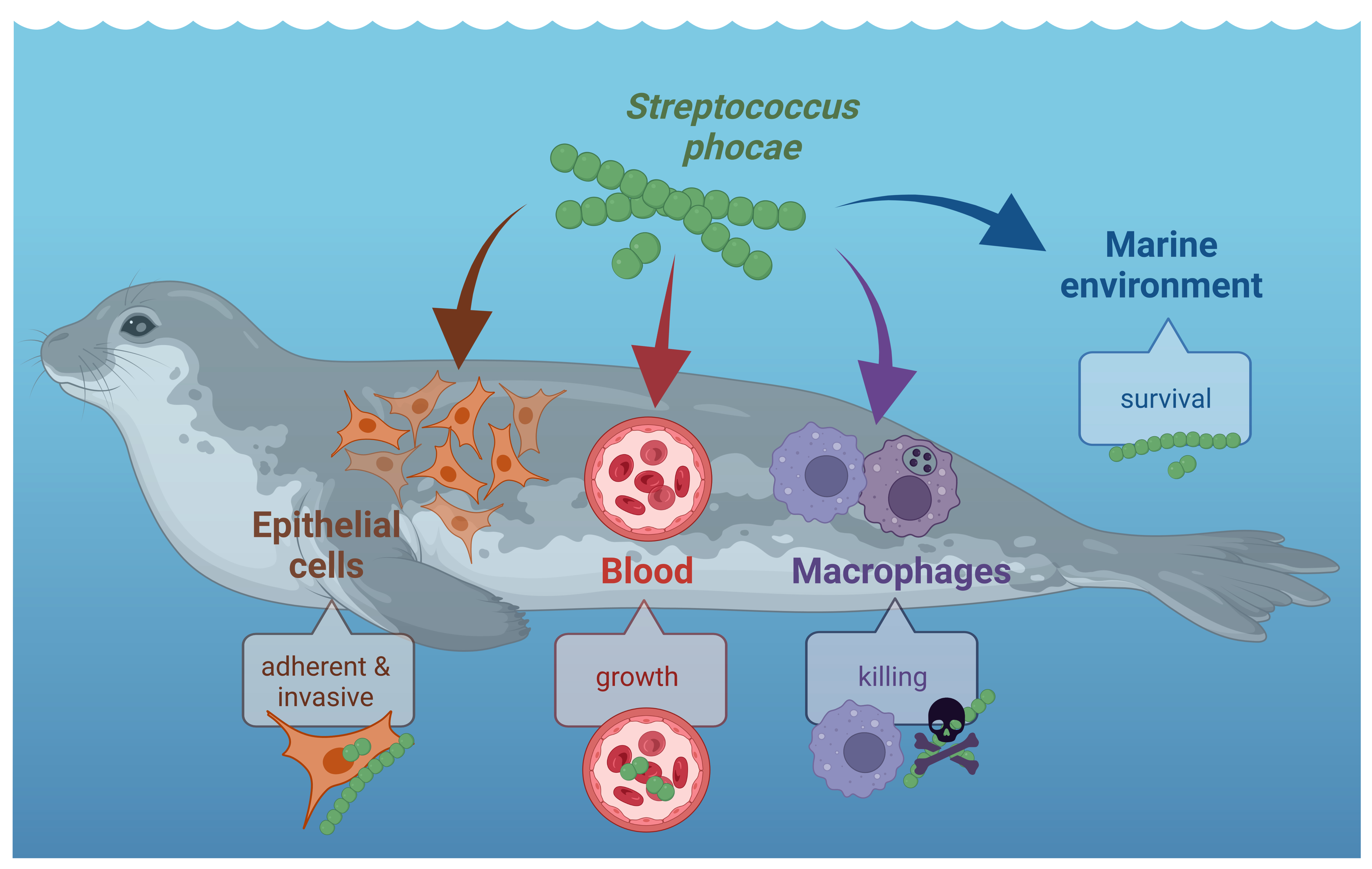

Supplement: S1 Graphical abstract — (TIF) [file pone.0296368.s003.tif]
